# Emotional Valence, Interdependence, and Job Autonomy as Predictors of Creativity Through Perspective-Taking: An Integrative Model

**DOI:** 10.3390/bs15030284

**Published:** 2025-02-28

**Authors:** Kyueun Han, You Jin Kim

**Affiliations:** 1 College of Kyedang General Education, Sangmyung University, Seoul 03016, Republic of Korea; 2 Department of Management, College of Business, City University of Hong Kong, Hong Kong; y.kim@cityu.edu.hk

**Keywords:** creativity, emotional valence, perspective-taking, interdependence, autonomy

## Abstract

This study examines the underexplored intersection of emotional valence and perspective-taking in workplace creativity, and how job characteristics like interdependence and autonomy moderate these relationships. Participants (*N* = 307; 41% women) recruited through Amazon’s Mechanical Turk platform and employed across various U.S. companies completed an experimental study where they were randomly assigned to recall either positive or negative workplace relationships. Through this manipulation, the participants identified specific colleagues with whom they had direct working experience and reported their emotional valence toward these relationships before completing questionnaires on perspective-taking, creativity, autonomy, and interdependence. Integrating emotional valence and perspective-taking into a moderated mediation model yielded insights into how these variables shape creativity within organizations. The findings demonstrate that positive emotional states significantly enhance creativity through perspective-taking, especially in environments that promote collaboration and independent decision-making. This research broadens workplace dynamics by illuminating the roles of emotional and contextual factors in fostering creativity. It provides practical implications for organizations, recommending positive emotional climates and roles that balance interdependence with autonomy to maximize employee creativity. This study’s comprehensive approach provides a holistic understanding of conditions that foster creativity in organizational environments, expanding on existing frameworks.

## 1. Introduction

The role of workplace relationships in organizational outcomes has garnered considerable attention (Ferris et al., 2009). These relationships involve exchanges between interacting members to achieve common goals (Ferris et al., 2009). Affect, a fundamental component of workplace relationships, has been widely examined, with these relationships typically conceptualized along a continuum ranging from positive to negative. However, existing studies have primarily focused on its influence on job satisfaction and performance, with limited exploration of its role in workplace creativity, which this study aims to address, as highlighted by Methot et al. (2016). Positive and negative affect—often characterized as liking and disliking, respectively—are well-established concepts in psychology (Rivera et al., 2024) and organizational studies (Casciaro & Lobo, 2008). Liking can significantly influence interpersonal characteristics and the longevity of workplace relationships (Nicholson et al., 2001).

Labianca et al. (1998) proposed a continuum of workplace relationships from friendship to avoidance. Casciaro and Lobo (2008) defined positive working relationships as those where individuals enjoy working together, experience pleasant interactions, and feel energized. Conversely, negative relationships involve unpleasant interactions and a lack of enjoyment in collaboration. Positive working relationships are crucial for fostering a productive workplace environment and ensuring quality workplace interactions (Lagman, 2024). Additionally, workplace happiness has been identified as a moderator in employee engagement and burnout, further strengthening the case for fostering positive relationships at work (Erer & Savaş, 2025).

Positive relationships are linked to favorable outcomes, including enhanced task performance, higher goal setting, and improved negotiation (Van Kleef et al., 2010), as well as higher performance ratings (Lefkowitz, 2000), increased organizational citizenship behavior (Allen & Rush, 1998), and better leader–member exchange quality (Wayne et al., 1997). These factors contribute to the overall employee health and well-being (Danna & Griffin, 1999). Employees who have deeper emotional bonds with their colleagues develop a stronger connection to their organization and experience more positive work-related outcomes (Ehrhardt & Ragins, 2019). Caring and respectful leader–member relationships promote meaningful work engagement, enhancing creativity (Stephens & Carmeli, 2017). The practical implications of understanding how emotional valence influences creativity are significant for modern organizations. Research suggests that positive emotions enhance workplace creativity, leading to improved problem-solving and innovation (Diener et al., 2020). Additionally, fostering positive emotions at work contributes to collaborative engagement and creativity in team settings (J. S. Yang & Hung, 2015). Empirical evidence from companies such as Google and IDEO suggests that fostering a positive emotional climate enhances employees’ ability to generate innovative solutions, underscoring the practical implications of emotional valence in organizational settings (Li et al., 2023). However, empirical findings on the relationship between positive workplace relationships and creativity have been mixed. Some studies suggest that positive relationships enhance creativity by fostering psychological safety and open communication, while others indicate that excessive cohesion may lead to conformity, thereby stifling innovative thinking. This inconsistency highlights the need for further investigation into potential moderating factors. High interpersonal cohesiveness improves creativity-related performance under high task cohesiveness but worsens performance under low task cohesiveness (Craig & Kelly, 1999).

This study’s primary objective is to explore the relationship between workplace relationships and individual creativity through the lens of motivational information processing. This approach addresses the inconsistent literature findings by proposing two key mechanisms. The first is the mediating role of perspective-taking. Motivated information processing theory posits that individuals engage in systematic and detailed information processing based on their epistemic motivation—the drive to understand the world accurately (Van Kleef et al., 2010). It is situationally dependent, increasing when tasks are perceived as attractive or personally involving and decreasing when they are uninteresting (Webster et al., 1996). It was hypothesized that individuals are more likely to perceive tasks as attractive and engaging when working with colleagues with whom they have positive relationships, leading to higher epistemic motivation.

Higher epistemic motivation enhances attention, categorization, recall, and information integration, which are essential for creativity. It also reduces reliance on stereotypes, decreases prototypical information search (Kruglanski & Mayseless, 1988), and increases systematic information processing (De Dreu et al., 2008). Consequently, epistemically motivated employees are more likely to focus on novel ideas. Creativity requires attention to both novelty and usefulness. Other-focused knowledge sharing fosters novel and useful idea generation—the cornerstones of creativity and innovation (Grant & Berry, 2011). Perspective-taking can engender this focus on usefulness by encouraging the consideration of diverse viewpoints and a better understanding of original and practical ideas (De Dreu et al., 2008; Grant & Berry, 2011). Therefore, it is hypothesized that positive workplace relationships promote perspective-taking, generating creative and useful ideas.

Perspective-taking thus plays a crucial role in fostering individual creativity by enabling individuals to adopt alternative perspectives, enhance cognitive flexibility, and engage in deeper problem-solving processes. By facilitating knowledge exchange and integrating diverse viewpoints, perspective-taking promotes novel idea generation and the refinement of innovative concepts through iterative feedback and collaborative synthesis. However, an in-depth analysis of this theorized linkage and new theoretical perspectives and empirical investigations are needed to deepen the understanding of how the emotional valence of workplace relationships (i.e., positive versus negative) is associated with individual creativity through perspective-taking. The connection between emotional valence and creativity is more evident in specific workplace outcomes. When employees experience positive emotional relationships, they are more likely to engage in creative behaviors such as suggesting process improvements, developing innovative product ideas, and proposing novel solutions to organizational challenges (Madrid & Patterson, 2025). This relationship becomes particularly vital in knowledge-intensive industries where creative outputs significantly influence organizational performance.

The second mechanism involves the situational context, which helps shape workplace behavior (Johns, 2006). Amabile et al. (1996) emphasized the importance of work environment in influencing creativity. According to motivated information processing theory, the work environment affects epistemic motivation (De Dreu et al., 2008). Individuals exhibit higher epistemic motivation when they perceive issues as highly involving, compared to when they view them as less involving, or when they feel more accountable for their decisions). It was proposed that personal epistemic motivation influences perspective-taking during interpersonal workplace interactions, particularly when individuals feel involved with their colleagues in tasks or responsible for their judgments and decisions. In contrast, when tasks are independent or when employees have little discretion to share knowledge owing to a structured work system, the emotional valence of workplace relationships may not influence perspective-taking.

This study proposes and tests the interaction between the emotional valence of working relationships and job characteristics in predicting perspective-taking and creativity. To clarify the predictors of creativity, this study develops a moderated mediation model with perspective-taking as a mediator and task interdependence and job autonomy as moderators.

This study first delineates the mechanisms through which positive and negative workplace relationships influence perspective-taking, emphasizing their differential effects on cognitive and social processing. Then, it addresses how job characteristics, particularly task interdependence and job autonomy, moderate the relationship between workplace relationships and perspective-taking, leading to creativity. The final hypothesis integrates these ideas into a moderated mediation model, in which the interaction between the emotional valence of working relationships and job characteristics predicts creativity, mediated by perspective-taking. The results are presented from an experimental study that tested the current hypotheses.

## 2. This Study

### 2.1. Emotional Valence of Working Relationships and Perspective-Taking

Positive working relationships are characterized by trust and a willingness to cooperate (Van Kleef et al., 2010). Trust not only fosters cooperative behaviors but also facilitates perspective-taking by creating a psychologically safe environment where individuals feel comfortable considering others’ viewpoints. This openness to different perspectives enhances collaboration and problem-solving in the workplace, reinforcing the role of trust in effective teamwork and decision-making. This openness to different perspectives enhances collaboration and problem-solving in the workplace. Trust, particularly its affective dimension, is crucial in fostering cooperative behaviors and enhancing work performance (McAllister, 1995). Affective trust, developed through interactions, enhances work performance by increasing need-based monitoring and citizenship behavior (McAllister, 1995). Van Kleef et al. (2010) reviewed research supporting the idea that positive affect, such as happiness, promotes liking, affiliation, trust, and cooperation (Barsade, 2002; George, 1995; Lazarus, 1991; Staw et al., 1994). Individuals prefer working with those who exhibit positive affect (Lazarus, 1991). A leader’s positive affect is positively associated with job satisfaction, job involvement, and group performance in service settings (George, 1995). Positive affect enhances work performance and social support within work relationships (Staw et al., 1994).

In experimental settings, introducing a happy confederate has been shown to foster positive working relationships, which, in turn, enhance cooperation, reduce conflict (Barsade, 2002), and strengthen long-term professional trust and collaboration (Nicholson et al., 2001). This manipulation highlights the role of emotional valence in shaping workplace interactions and its implications for team dynamics.

Individuals are more likely to engage in prosocial behaviors toward colleagues with whom they share positive affect (Aliane & Alshiha, 2025). This effect is particularly evident in experimental settings, where emotional contagion and shared positive emotions within teams have been shown to enhance perspective-taking and collaborative outcomes such as knowledge sharing. For example, leaders with positive affect are associated with increased prosocial behaviors at work. This study posits that a worker’s positive mood fosters positive working relationships, which induce perspective-taking through other-focused psychological processes. Consistently, positive work relationships that evoke positive affect facilitate other-focused organizational spontaneity, such as helping co-workers or making constructive suggestions (George & Brief, 1992). Recent studies have highlighted how emotional contagion and shared positive emotions within teams enhance perspective-taking and collaborative outcomes such as knowledge sharing (Xu et al., 2024).

A leader’s humility, which is closely linked to positive affect, enhances perspective-taking abilities in organizations by fostering an open and inclusive communication environment. This contributes to the broader framework of positive workplace interactions, reinforcing the role of emotional valence in shaping trust and cooperation by fostering an open and inclusive communication environment. Humble leaders acknowledge their limitations and value the perspectives of others, which encourages employees to engage in mutual learning and cognitive flexibility. This, in turn, enhances perspective-taking by reinforcing a culture of shared knowledge and respect for diverse viewpoints (Wang et al., 2017). Additionally, authenticity in teams, combined with a positive emotional climate and strong perspective-taking, enhances team performance by fostering the integration of unique perspectives; however, it can hinder collaboration if these perspectives are not heard or valued (Leroy et al., 2021).

Building on this, individuals may be more likely to consider the perspective of a colleague with whom they have a positive working relationship, as the trust and cooperative intent cultivated through positive affect can create a conducive environment for perspective-taking, owing to increased trust, a greater intention to cooperate, and a higher likelihood of engaging in prosocial behaviors. They are also less inclined to take the perspective of a colleague with whom they have a negative relationship.

**Hypothesis** **1.** *Individuals are more likely to take the perspective of a colleague with whom they have a positive working relationship than one with whom they have a negative relationship.*

### 2.2. Role of Perspective-Taking in Creativity and the Mediating Effect of Emotional Valence

Perspective-taking can lead to a range of positive outcomes at work, such as cooperative or helping behaviors within teams (Parker & Axtell, 2001) and increased creativity (Grant & Berry, 2011). Specifically, by adopting others’ viewpoints, employees can enhance their ability to generate innovative solutions and contribute to a more collaborative and adaptive work environment (Zhao et al., 2024). According to motivated information processing theory, individuals generate useful information when they consider others’ perspectives (De Dreu et al., 2008). Other-focused psychological processes heighten attention to others’ needs, fostering prosocial behaviors and creativity (Grant, 2007).

Grant and Berry (2011) reported that employees are motivated to produce novel and useful ideas when they consider others’ perspectives, which enhances their creative output. This aligns with the motivated information processing theory (De Dreu et al., 2008; Grant & Berry, 2011) and creative cognitive processing theory (Parker & Axtell, 2001), which suggest that taking co-workers’ or supervisors’ perspectives helps create useful ideas that benefit organizational members beyond self-interest, ultimately enhancing individual workplace creativity.

Effective communication, awareness of team direction, understanding of members’ roles, and mutual support through perspective-taking—key characteristics of shared leadership—can significantly enhance creativity by facilitating the integration of diverse viewpoints and promoting shared problem-solving (Ali et al., 2023). This highlights the role of perspective-taking not just as an individual cognitive process but as a collective mechanism that strengthens team-based creativity. Additionally, organizations can more effectively encourage workplace creativity by balancing novelty and usefulness through perspective-taking and combining extrinsic, intrinsic, and prosocial motivations (Berry, 2020).

Although perspective-taking generally enhances creativity, an over-reliance on others’ viewpoints may potentially inhibit certain aspects of creative expression. Research suggests that placing too much emphasis on perspective-taking could lead to conformity bias, causing individuals to prioritize alignment with others’ opinions rather than producing genuinely original ideas (Z. Yang & Hung, 2021). This excessive focus on others’ perspectives might result in creative inhibition due to heightened self-consciousness and reduced cognitive autonomy (Gunia et al., 2024). Furthermore, continuous attention to others’ viewpoints might deplete cognitive resources needed for innovative thinking, potentially undermining the novelty dimension of creativity (Fasbender et al., 2024).

The relationship between perspective-taking and creativity thus appears to exhibit an inverted U-shaped pattern, where moderate levels of perspective-taking optimize creative output by striking a balance between novelty and usefulness (Hundschell et al., 2022). This pattern aligns with findings that excessive perspective-taking can inhibit originality due to conformity bias and cognitive overload while insufficient perspective-taking limits the integration of diverse viewpoints (Reiter-Palmon et al., 2024). By maintaining a moderate level, individuals can benefit from the advantages of perspective-taking without its potential drawbacks. This optimal balance allows individuals to leverage insights from others for practicality while preserving their original ideas. Organizations should therefore encourage perspective-taking while also fostering an environment that supports individual creative autonomy (Park & Kang, 2024).

Evidence suggests that perspective-taking can mediate the relationship between the emotional valence of working relationships (e.g., positive/negative) and individual creativity. Perspective-taking leads to individual creativity, and individuals are more likely to take the perspective of a colleague when they have a positive working relationship than when they have a negative one.

A positive relationship evokes positive affect, which builds trust and affiliation, leading individuals to consider and take others’ perspectives (Jatoi et al., 2024). This process, in turn, enhances creativity by encouraging cognitive flexibility, fostering novel idea generation, and facilitating the integration of diverse viewpoints into practical solutions. Thus, perspective-taking likely acts as a mediator that translates the emotional valence of working relationships into individual creativity.

**Hypothesis** **2.** *Perspective-taking positively affects individual creativity.*

**Hypothesis** **3.** *Perspective-taking mediates the relationship between the emotional valence of working relationships and individual creativity.*

### 2.3. The Moderating Role of Interdependence and Autonomy in the Indirect Effect of Emotional Valence on Creativity via Perspective-Taking

Affect plays a crucial role in working relationships; however, research in this area is often constrained by existing theoretical frameworks and a lack of studies adopting a multilevel perspective. Research also suggests that the structure of the task environment influences organizational behavior and performance outcomes (Gonzalez, 2024). This aligns with Amabile et al.’s (1996) theory that work characteristics significantly impact creativity. Furthermore, studies highlight that individual skill differences and the autonomy provided within the work environment are key determinants of creative engagement and problem-solving effectiveness (Lettner, 2024). This study suggests that emotional valence in working relationships interacts with work environments to influence work behaviors, particularly individual creativity. Intimate relationship theory suggests a positive relationship between the emotional valence of working relationships and perspective-taking, as members consider the perspectives of those they emotionally favor (Micheli et al., 2024). Positive relationships facilitate open information flow, allowing individuals to better understand their colleagues’ needs and desires and the best way to assist them (Gaur & Ebrahimi, 2013). However, if the task is highly independent or if the work system is rigidly structured, limiting the discretion to share knowledge or information, the emotional valence may have less influence on perspective-taking. This study proposes that the nature of this relationship varies depending on specific work characteristics, particularly task interdependence and job autonomy.

#### 2.3.1. Task Interdependence

Task interdependence refers to the reliance on others to complete work tasks (Kiggundu, 1981; Morgeson & Humphrey, 2006) and functions as a team characteristic (Campion et al., 1993; Van der Vegt et al., 2001) and an individual job characteristic (Kiggundu, 1981) affecting job satisfaction, creativity, and innovative behavior (Gilson & Shalley, 2004; Van der Vegt & Janssen, 2003).

Interdependence moderates the relationship between cohesion and performance (Gully et al., 1995; Liden et al., 2006). Task interdependence moderates the relationship between leader–member exchange differentiation and performance, with a higher task interdependence leading to greater differentiation among group members, improving performance (Liden et al., 2006). Therefore, this study speculates that higher task interdependence will enhance communication, cooperation, and harmonized behavior, increasing perspective-taking. It is expected that interdependence will moderate the relationship between the emotional valence of working relationships and perspective-taking, with a more pronounced effect in high-task-interdependence situations.

#### 2.3.2. Autonomy

Job autonomy—the degree of freedom, independence, and discretion in work (Morgeson & Humphrey, 2006)—influences how individuals engage with their colleagues. Morgeson and Humphrey (2006) classified job autonomy into work scheduling, decision-making, and work methods. Given this study’s focus on interpersonal working relationships, decision-making and work method autonomies are particularly relevant.

Autonomy leads to positive outcomes such as job satisfaction, work motivation, and effectiveness (Zinovieva & Mengov, 2025). It also enhances information sharing (Cabrera et al., 2006), facilitating knowledge exchange and collaboration within organizations. High job autonomy encourages individuals to engage in informal interactions and share insights with colleagues, especially those with whom they have positive working relationships (Lucena Barbosa & Borges-Andrade, 2022). Conversely, low autonomy, in which tasks are more structured and routine, diminishes the opportunity for such interactions, reducing the likelihood of perspective-taking. Consequently, this study proposes that positive working relationships will have a stronger influence on perspective-taking in high-autonomy situations than in low-autonomy ones.

**Figure 1:**
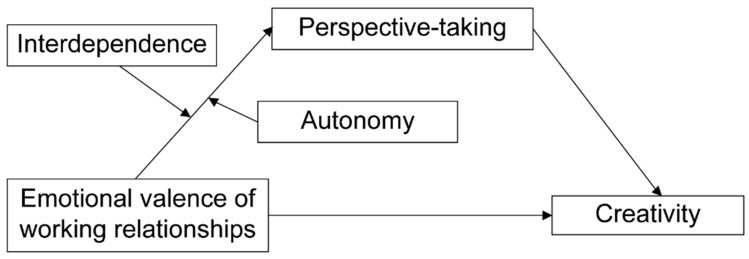
Hypothesized model.

The theoretical framework outlined above suggests several testable relationships between emotional valence, perspective-taking, and creativity in organizational settings (e.g., Han et al., 2022). Specifically, this study proposes that the relationship between the emotional valence of working relationships and creativity is bounded by job characteristics and mediated by perspective-taking. We posit a moderated mediation model in which interdependence and autonomy function as first-stage moderators, meaning they influence the extent to which emotional valence affects perspective-taking. This, in turn, predicts individual creativity by shaping the way individuals interpret and respond to emotional cues in their working relationships (Figure 1).

To empirically examine these relationships and their contextual constraints, we designed a comprehensive study that captures both the emotional and structural aspects of workplace interactions. Our research design specifically addresses how positive versus negative workplace relationships influence perspective-taking and creativity, while investigating the moderating effects of task interdependence and job autonomy. This approach allows us to examine both the direct relationships between variables and the broader mechanisms underlying workplace creativity, ensuring a comprehensive understanding of how these factors interact.

The hypothesized relationships between these variables necessitate a methodological approach that can effectively capture both individual psychological processes and organizational contextual factors. Additionally, our interest in examining how job characteristics moderate the relationship between emotional valence and perspective-taking requires the careful consideration of measurement approaches that can assess these complex interactions. Therefore, we employed a multi-measure design that incorporates validated scales for assessing emotional valence in workplace relationships, perspective-taking tendencies, creative output, and job characteristics.

Building on this theoretical foundation, we now present our research methodology, designed to rigorously test our hypothesized model and account for potential alternative explanations. This section details our sample selection, data collection procedures, and analytical approach, aligning our methodological choices with our theoretical framework and research objectives. The following section outlines our sample selection, data collection procedures, and analytical approach, demonstrating how our methodological choices align with our theoretical framework and research objectives.

**Hypothesis** **4.** *Interdependence and autonomy moderate the indirect effect of emotional valence on creativity via perspective-taking.*

## 3. Materials and Methods

### 3.1. Participants

The sample size was determined by a power analysis using GPower 3.1. Assuming a small effect size (f = 0.15), alpha level of 0.05, and desired power level of 0.80, a minimum sample size of 220 was required. Three hundred and seven employees were recruited via a Human Intelligence Task (HIT) posted on MTurk, a research service that matches social science researchers with an international sample willing to participate in online surveys. Eligible participants had to be working for an organization in the United States and have another direct co-worker. Participants were 41% women, with the following ethnicities: 58% Asian, 26.4% White, 6% Black, 4% Hispanic, and 5.6% other/did not report. They were informed that they could withdraw from the study at any time without penalty and received compensation of USD 0.40 for their involvement.

### 3.2. Procedures

The participants answered manipulation checks and completed questionnaires on perspective-taking (Davis et al., 1996), creativity (George & Zhou, 2001), and autonomy and interdependence (Morgeson & Humphrey, 2006). Two safeguard questions were included to check whether they carefully read the questions and answered them. Of the 405 employees who responded to the questionnaire, 97 did not answer these two questions correctly and were eliminated, resulting in a final sample of 307. After providing informed consent, participants were randomly assigned to one of two conditions: (1) positive and (2) negative working relationship with an employee.

### 3.3. Manipulations

The emotional valence of the working relationship was manipulated by asking participants to identify an employee—by first and last name—with whom they had directly worked and to whom they felt either positive (or negative) emotions. They then completed questionnaires regarding the emotional valence of working relationships with the identified person.

### 3.4. Measures

This study used previously validated scales for all focal variables (1 = to a very small extent; 7 = to a very large extent).

#### 3.4.1. Manipulation Checks

Participants indicated the degree to which they agreed regarding the relationship with the identified person using four items (Casciaro & Lobo, 2008), including “(the name of the identified person) is enjoyable to work with”, “I personally like this individual”, and “When I interact with this person, I feel energized” (α = 0.96).

#### 3.4.2. Perspective-Taking

Participants indicated the extent to which they took the identified person’s perspectives using a four-item scale (Grant & Berry, 2011), including “On the job, I frequently try to take the person’s perspectives” and “At work, I regularly seek to understand others’ viewpoints” (α = 0.91).

#### 3.4.3. Creativity

This study measured creativity with a thirteen-item scale (George & Zhou, 2001), including “When I work with (the name of the identified person), I exhibit creativity on the job when given the opportunity to” and “When I work with (the name of the identified person), I develop adequate plans and schedules for the implementation of new ideas” (α = 0.95).

#### 3.4.4. Interdependence

This study measured interdependence using six items (Morgeson & Humphrey, 2006), including “The job requires me to accomplish my job before others complete their job” (initiated interdependence) and “The job activities are greatly affected by the work of other people” (received interdependence) (α = 0.80).

#### 3.4.5. Autonomy

This study measured autonomy using six items (Morgeson & Humphrey, 2006), including “The job gives me a chance to use my personal initiative or judgment in carrying out the work” (decision-making autonomy) and “The job allows me to make decisions about what methods I use to complete my work” (work method autonomy) (α = 0.90).

### 3.5. Research Procedures and Data Processing

First, participants were told about the study’s purpose and then asked to provide written informed consent (which stated that the survey was anonymous and only for academic research and that all information would be confidential). After reading the instructions, participants independently completed the online questionnaire and submitted their responses. This study was approved by the Ethics Committee of Yonsei University (no. 1040917201503SB12502).

The data were sorted and analyzed using SPSS 28.0 and the PROCESS v4.2 macro (Hayes, 2013). The program can verify various mediated moderation and moderated mediation models based on the deviation-corrected percentile bootstrap method. By taking 5000 bootstrap samples (sample size = 307), the robust standard error and bootstrap confidence interval (CI) of parameter estimation were obtained. The result was considered significant if the CI did not contain 0 (Erceg-Hurn & Mirosevich, 2008). Model 4 of PROCESS was used to test the mediating effect of perspective-taking. To test the moderated mediation effects of interdependence and autonomy on the relationship between emotional valence and perspective-taking, as well as the subsequent impact on creativity, a moderated mediation analysis was conducted using Model 9 (Hayes, 2017) with 5000 bootstrapped samples.

## 4. Results

### 4.1. Manipulation Checks

The participants reported better working relationships in the positive condition compared to those in the negative condition (t(305) = 22.04, *p* < 0.01, d = 2.52; M_negative_ = 2.96; M_positive_ = 5.87).

### 4.2. Descriptive Statistics of and Correlation Between the Variables

Table 1 presents the descriptive statistics of the emotional valence of working relationships, perspective-taking, creativity, interdependence, and autonomy. Significant positive correlations were observed between the following variables: emotional valence and perspective-taking (r = 0.52, *p* < 0.001); emotional valence and creativity (r = 0.41, *p* < 0.001); and perspective-taking and creativity (r = 0.63 (*p* < 0.001). The correlation coefficients between any two of these three variables did not exceed 0.8, indicating a low-to-moderate correlation and no collinearity (Cohen et al., 2009).

### 4.3. The Mediating Role of Perspective-Taking

**Figure 2:**
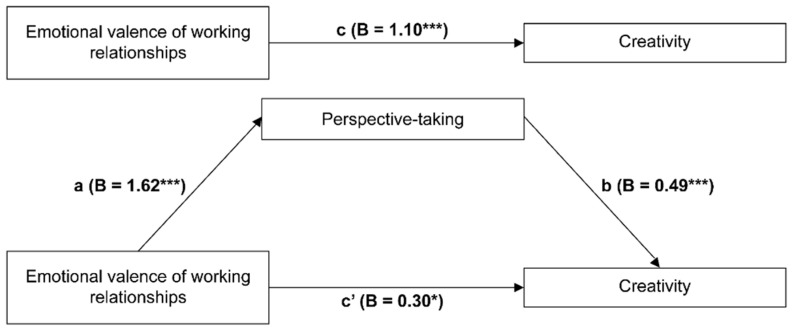
Mediating role of perspective-taking in the relationship between emotional valence of working relationships and creativity. * *p* < 0.05, *** *p* < 0.001. Path a (β = 1.62 ***): The effect of the independent variable (Emotional valence of working relationships) on the mediator (Perspective-taking); Path b (β = 0.49 ***): The effect of the mediator (Perspective-taking) on the dependent variable (Creativity); Path c (β = 1.10 ***): The total effect of the independent variable on the dependent variable; Path c′ (β = 0.30 *): The direct effect of the independent variable on the dependent variable when controlling for the mediator.

In Model 1, emotional valence significantly and positively predicted creativity (B = 1.10, t = 7.88, *p* < 0.001). In Model 2, emotional valence significantly and positively predicted perspective-taking (B = 1.62, t = 10.66, *p* < 0.001). In Model 3, perspective-taking as a mediator significantly and positively predicted creativity (B = 0.49, t = 11.12, *p* < 0.001), supporting Hypothesis 2. Emotional valence significantly and positively predicted creativity (B = 0.30, t = 0.03, *p* = 0.031); however, its predictive power in Model 3 was weaker than that in Model 1, which suggests that perspective-taking partially mediates the effect of emotional valence on creativity, supporting Hypothesis 3 (Figure 2). A bias-corrected nonparametric percentile bootstrapping analysis indicated an indirect effect of 0.80 and a 95% confidence interval (CI) of 0.60–1.02, which excludes 0. The mediating effect accounted for 72.82% of the total effect, indicating a significant mediating effect of perspective-taking (Table 2).

### 4.4. The Moderated Mediating Effects of Interdependence and Autonomy on the Relationship Between Emotional Valence and Creativity Through Perspective-Taking

**Figure 3:**
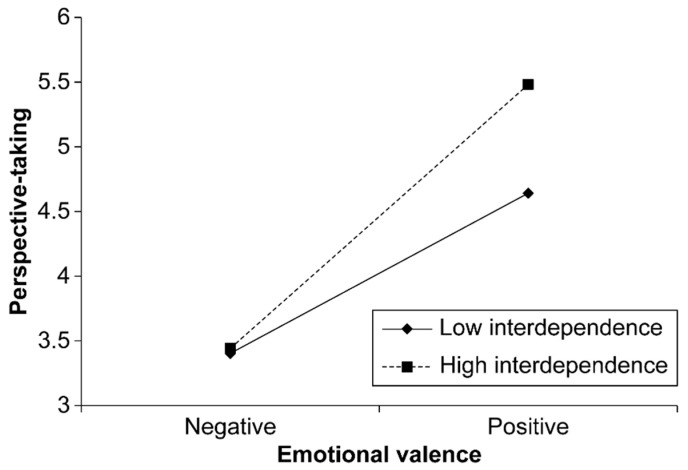
Moderating effect of interdependence on the relationship between emotional valence and perspective-taking.

**Figure 4:**
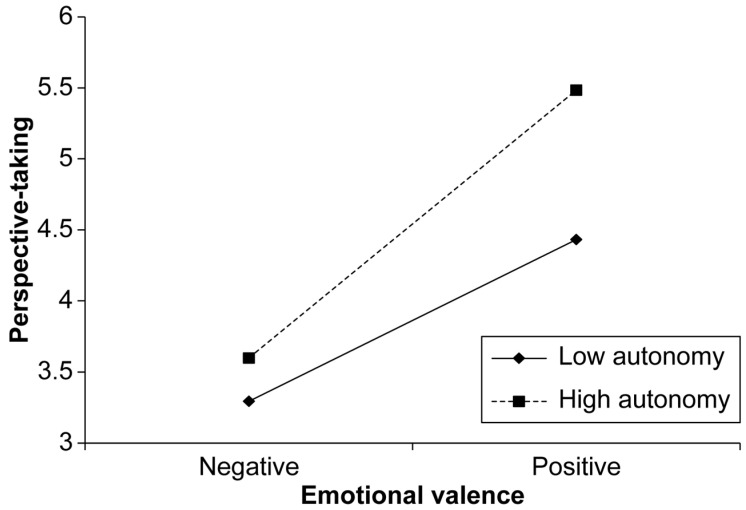
Moderating effect of autonomy on the relationship between emotional valence and perspective-taking.

The interaction between emotional valence and interdependence significantly predicted perspective-taking (B = 0.35, SE = 0.14, *p* = 0.011), suggesting that interdependence moderates the relationship between emotional valence and perspective-taking. The positive impact of emotional valence on perspective-taking was amplified at higher levels of interdependence (Figure 3). Similarly, the interaction between emotional valence and autonomy was significant (B = 0.30, SE = 0.14, *p* = 0.031), indicating that autonomy also moderates this relationship. The positive impact of emotional valence on perspective-taking was amplified at higher levels of autonomy (Figure 4). The conditional effects of emotional valence on perspective-taking were examined at different levels of interdependence and autonomy (Table 3). At higher levels of interdependence (5.83) and autonomy (6.00), emotional valence had a more pronounced positive effect on perspective-taking (B = 2.12, SE = 0.22, *p* < 0.001), demonstrating the strong moderating influence of these factors.

Table 4 presents the analysis of the indirect effects of emotional valence on creativity through perspective-taking, moderated by interdependence and autonomy. Emotional valence had a significant direct effect (B = 0.30, SE = 0.14, *p* = 0.030) and perspective-taking had a significant positive effect on creativity (B = 0.49, SE = 0.04, *p* < 0.001). The partial moderated mediation indices showed significant effects for both interdependence (index = 0.17, BootSE = 0.07, lower limit confidence interval (BootLLCI) = 0.03, upper limit confidence interval (BootULCI) = 0.32) and autonomy (index = 0.15, BootSE = 0.07, BootLLCI = 0.01, BootULCI = 0.31); the limits of the 95% bootstrap CI did not contain zero, confirming the mediation of interdependence and autonomy.

The indirect effects of emotional valence on creativity were most pronounced under high interdependence and autonomy conditions (5.83 and 6.00, respectively), with an effect size of 1.05 (BootSE = 0.16, BootLLCI = 0.74, BootULCI = 1.38), indicating that the pathway from emotional valence to creativity through perspective-taking is significantly enhanced by high interdependence and autonomy.

## 5. General Discussion

This study presents strong evidence that emotional valence in workplace relationships significantly affects creativity, with perspective-taking serving as a crucial mediator. Importantly, this relationship is moderated by interdependence and autonomy, highlighting the complex interaction between individual emotions and the organizational environment. These findings provide a comprehensive perspective on how emotional and contextual factors interact to foster creativity in organizational settings.

Previous research has demonstrated that positive workplace relationships contribute to various organizational outcomes, including task performance, motivation, goal setting, and negotiation (Baron, 1990; Staw et al., 1994; Van Kleef et al., 2010), as well as organizational citizenship behavior (Miles et al., 2002), job satisfaction (Weiss et al., 1999), higher performance ratings (Lefkowitz, 2000), and employee health and well-being (Danna & Griffin, 1999). However, the influence of emotional valence on creativity remains relatively understudied.

This study reveals a complex relationship between emotional valence and creativity, with perspective-taking acting as a mediator and interdependence and autonomy functioning as moderators. It illustrates how positive emotions enhance creativity by fostering perspective-taking, particularly in environments that encourage collaboration and autonomous decision-making. These insights expand our understanding of how workplace dynamics influence creative outcomes and offer a deeper comprehension of the conditions conducive to creativity.

This study makes several theoretical contributions by integrating emotional valence and perspective-taking into a moderated mediation model. First, it demonstrates how emotional experiences and perspective-taking interact with job characteristics to influence creativity, thereby contributing to creativity theory. Akaki and Maeno (2024) found that feedback positively influences a team’s creative behavior, mediated by conflict and idea acceptance, while conflict negatively moderates creativity during idea generation. This aligns with the present study’s findings on emotional valence and perspective-taking, as both suggest that managing social and emotional dynamics within teams is crucial for fostering creativity. These results highlight the importance of managing conflict and fostering idea acceptance to maximize creative potential in collaborative environments. Second, the moderated mediation model provides a more nuanced understanding of how job characteristics influence this process.

Prior research on the relationship between emotional ambivalence and creativity suggests that emotional complexity may foster creative processes under certain conditions (Fong, 2006; Tyng et al., 2017). Studies examining the connection between prosocial motivations and creativity have highlighted that perspective-taking can amplify the positive effects of emotional states on creative outcomes (Grant & Berry, 2011).

In this regard, this research emphasizes the importance of considering both individual and contextual factors in theoretical models of creativity, offering a holistic perspective on the antecedents of creative behavior. Prior research has highlighted the role of emotions in enhancing creativity, particularly in environments that emphasize corporate social responsibility and employee well-being (George & Zhou, 2001; Morgeson & Humphrey, 2006). Furthermore, affect plays a role in working relationships—a factor that has been relatively underexplored in organizational research.

The findings suggest that creative individuals can benefit from positive relationships among colleagues, which help mitigate the negative aspects of the creative process (Füller et al., 2011). Furthermore, this study identified task interdependence and job autonomy as critical boundary conditions that shape the relationship between emotional valence, perspective-taking, and creativity. Positive working relationships encourage customized knowledge sharing in environments with high task interdependence and autonomy, which fosters individual creativity through perspective-taking (Gagné et al., 2019). This demonstrates how positive emotions between colleagues can activate the process of identifying and addressing others’ needs, leading to creative idea generation when interdependence and autonomy are high.

Building on the previous discussion of individual-level creativity, the moderated mediation effects of interdependence and autonomy manifest distinctively across organizational contexts and industry sectors, highlighting the broader organizational implications of these relationships. In knowledge-intensive industries, where both autonomy and interdependence are typically high, emotional valence demonstrates particularly strong effects on creativity through perspective-taking mechanisms (Liu et al., 2011). These dynamics present differently in traditional manufacturing or service industries, where standardization often constrains autonomy, suggesting the need for the deliberate creation of autonomous spaces to leverage the creative benefits of positive workplace relationships (Balkin et al., 2015).

Building on the discussion of creativity interventions, this research extends existing theoretical frameworks in several important ways by highlighting both practical and theoretical contributions. First, it advances the understanding of workplace creativity by integrating emotional valence theory with perspective-taking mechanisms, providing a more nuanced theoretical model of creative behavior in organizations. This integration clarifies the reasons behind the inconsistent findings in previous studies regarding workplace relationships and creativity, as it highlights the mediating role of perspective-taking and the specific conditions under which these relationships function.

Second, our findings contribute to the emotional valence literature by demonstrating that the source of emotional experiences matters significantly. While previous research has typically treated affect as a generalized state, our theoretical model differentiates between general emotional states and those that emerge specifically from workplace relationships. This distinction reveals that emotions rooted in relationships have unique implications for creative processes.

Third, this research advances motivated information processing theory by revealing how emotional valence influences perspective-taking within the context of workplace relationships. This theoretical contribution helps explain the psychological mechanisms through which interpersonal relationships affect knowledge processing and creative output.

Future research should explore other job characteristics, such as information processing, job complexity, and problem-solving, which might further qualify these relationships. This can help us better understand how various work environment elements interact with emotional valence and perspective-taking to influence creativity.

This study presents significant practical implications. Aligned with information processing theory (Simon, 1978), these findings indicate that the emotional valence of workplace relationships impacts creativity through perspective-taking in specific contexts. The interaction suggests that positive workplace relationships enhance perspective-taking, particularly when work interdependence or job autonomy is high.

The findings yield several concrete implications for organizations. Fostering creativity requires structured organizational support, integrating emotional valence and perspective-taking into a comprehensive framework (Khadpe et al., 2022). Organizations should implement cross-functional teams and job rotation to enhance perspective-taking and innovation. To sustain these benefits, recognition systems that reward creativity and collaboration are essential. Leadership training in emotional intelligence and active listening reinforces a self-sustaining cycle, strengthening positive workplace relationships and fostering creativity (Kour & Bhatia, 2025). Balanced workspaces and designated innovation periods facilitate collaboration while maintaining focus (Bouncken et al., 2021). Regular assessments of creativity, workplace relationships, and collaboration effectiveness ensure continuous improvement. Additionally, digital tools should be leveraged to enhance rather than replace interpersonal interactions, supporting relationship-building and maintaining a culture of creativity.

Thus, organizations should cultivate positive emotional climates and design roles that balance interdependence with autonomy. Such environments not only enhance employee creativity but also contribute to broader organizational innovation. This is consistent with Li and Liao’s (2017) study, which shows that help-giving in creative processes is positively associated with creativity when mediated by perspective-taking.

Organizations can enhance employee creativity by fostering positive emotional environments and encouraging perspective-taking. By promoting interdependence and autonomy, organizations can amplify the positive effects of emotional valence on creativity. These insights can inform organizational practices and policies aimed at cultivating a creative workforce, for instance, designing jobs that balance autonomy with collaborative opportunities and implementing training programs that enhance emotional intelligence and perspective-taking. Prior research has also emphasized the importance of emotional management and organizational justice in promoting happiness and creativity at work (Davis et al., 1996; Morgeson & Humphrey, 2006).

This study offers three key contributions to the existing creativity literature. First, while numerous studies have investigated the relationship between workplace affect and creativity (e.g., Amabile et al., 2005), most have conceptualized affect as a personal emotional state, regardless of its origin (e.g., Amabile et al., 2005; Brief & Weiss, 2002). This scope is narrowed to workplace relationships (e.g., Casciaro & Lobo, 2008) and examines how the emotional valence of these relationships influences creativity. For example, the impact of simply feeling happy on creativity at work may differ from that of feeling happy owing to a positive working relationship. Affect derived from workplace relationships may have a more substantial influence on creative tasks that involve knowledge sharing through interpersonal interactions. Moreover, Zhou and Hoever (2023) emphasized the importance of both positive and negative relational dynamics in workplace creativity, advocating for a more nuanced understanding of their interaction.

Second, this study delineates the relationship between positive and negative workplace relationships and creativity by illustrating the mechanism through which epistemic motivation leads to perspective-taking. Intrinsic motivation leading to prosocial perspective-taking promotes creativity (e.g., Anderson et al., 2014; Grant & Berry, 2011), and work practices such as integrated understanding, experiencing others’ jobs, and flexible role orientation encourage perspective-taking (Parker & Axtell, 2001). However, the psychological antecedents of such intrinsic motivation remain unclear. It is proposed that the emotional valence of workplace relationships can be a precursor to the motivation leading to creativity.

Third, the inconsistent findings regarding the role of positive workplace relationships in creativity are addressed by suggesting task interdependence and autonomy as boundary conditions. Positive workplace relationships promote creativity via perspective-taking only under high task interdependence and autonomy, filling a gap in the creativity literature. Additionally, Williams (2016) highlighted that leaders’ empathy and perspective-taking are critical in fostering a work environment conducive to creativity and organizational citizenship, aligning with the current findings.

The findings of this study present significant empirical implications for future research. The observed interaction between emotional valence and job characteristics underscores the necessity for future research to incorporate both relational and structural variables when examining workplace creativity. For instance, emotional valence may enhance perspective-taking in highly interdependent jobs, leading to more innovative problem-solving. Similarly, autonomy may moderate the impact of emotional valence, fostering creativity when individuals have greater decision-making freedom. The measurement methodologies established in this study, particularly those designed to assess the emotional valence of workplace relationships, provide validated tools for future empirical research.

Moreover, the findings challenge the conventional empirical perspective that conceptualizes creativity as a direct consequence of emotional states. Alternative perspectives suggest that creativity emerges from a combination of cognitive flexibility, environmental influences, and intrinsic motivation, rather than being solely driven by emotional states. Instead, the results indicate the necessity of employing more sophisticated empirical models capable of capturing the intricate interplay among emotions, perspective-taking, and job characteristics. These findings have methodological implications, suggesting that future research should adopt multilevel and longitudinal approaches, as multilevel models allow for the examination of individual, team, and organizational influences on creativity, while longitudinal designs help capture temporal variations and causal relationships within these dynamic interactions.

This study has some limitations. Self-reported measures may introduce bias, limiting causal interpretations. The sample may not fully represent the broader population, restricting the generalizability of the findings. Future research should use longitudinal or experimental designs to strengthen causal conclusions. Additionally, reliance on self-reported data for constructs such as emotion regulation and perspective-taking has been previously critiqued (Erceg-Hurn & Mirosevich, 2008).

Although this study positions emotional valence as a predictor, it does not establish causality between perspective-taking and creativity. While prior research suggests that perspective-taking predicts creativity (Grant & Berry, 2011), highly creative individuals may naturally engage in perspective-taking more frequently. As emotional valence in workplace relationships is dynamic (Lazarus, 2006), future studies should examine intra-individual fluctuations over time to gain deeper insights into its role in workplace creativity.

Our methodological approach warrants discussion. While the direct assessment of workplace relationships may seem ideal, our use of directed recall with random assignment provided key advantages. First, randomization is controlled for potential confounders. Second, it minimized social desirability bias that could arise from direct assessments, particularly in negative relationship contexts. However, this approach may primarily capture immediate emotional responses rather than long-term relationship valence. Future research should employ longitudinal studies to track workplace relationship evolution and its impact on creativity. Alternative methods, such as daily diary studies or experience sampling, could offer deeper insights into the sustained effects of emotional valence on creative processes (e.g., Sutu, 2022).

Future studies should consider multiple assessment methods, including peer ratings, observational data, and objective indicators of relationship quality. Triangulating these approaches would improve validity and provide a more comprehensive understanding of how workplace relationships influence creativity through perspective-taking. Additionally, examining moderating variables such as organizational culture or leadership styles could refine the broader applicability of these findings. Investigating these relationships in different cultural contexts would clarify their contextual relevance.

Moreover, future research could explore the long-term effects of emotional valence and perspective-taking on creativity, further clarifying their evolving interplay. Factors such as gaze direction and emotional context significantly impact social cognition, which is crucial for perspective-taking and creativity (Hayes, 2013, 2017). A holistic approach incorporating emotional and contextual factors is essential to understanding the psychological mechanisms underlying creativity (Williams et al., 2024). Cross-cultural and industry-specific research would refine our understanding of how emotional valence, perspective-taking, and job characteristics interact to shape creative outcomes.

By integrating emotional valence and perspective-taking into workplace creativity models, this study offers a novel framework for understanding the psychological mechanisms underlying innovation. These findings provide actionable insights for organizations aiming to cultivate a creative and emotionally intelligent workforce. Future research should further investigate how these mechanisms evolve over time and across cultural contexts, ultimately contributing to a more comprehensive theoretical and practical foundation for workplace creativity and innovation.

## Figures and Tables

**Table 1:** Means, standard deviations, and correlations among study variables.

Variables	Mean	SD	1	2	3	4	5
1. Emotional valence ^a^		0.51	(0.50)				
2. Perspective-taking	4.25	1.56	0.52 **	(0.91)			
3. Creativity	4.49	1.34	0.41 **	0.63 **	(0.95)		
4. Interdependence	4.81	1.07	−0.03	0.12 *	0.11 *	(0.80)	
5. Autonomy	5.21	1.08	0.17 **	0.29 **	0.42 **	0.16 **	(0.90)

Note: N = 307. Cronbach’s alphas are presented in parentheses along the diagonal. ^a^ Manipulated conditions coded as 0 = negative working relationship condition and 1 = positive working relationship condition. * *p* < 0.05, ** *p* < 0.01.

**Table 2:** Mediating effect of perspective-taking.

	Model 1			Model 2			Model 3		
	Creativity			Perspective-Taking			Creativity		
	B	SE	β	B	SE	β	B	SE	β
Emotional valence	1.10 ***	0.14	0.41	1.62 ***	0.15	0.52	0.30 *	0.14	0.11
Perspective-taking							0.49 ***	0.04	0.57
R2	0.17			0.27			0.41		
F	62.08 ***			113.64 ***			105.35 ***		

N = 307. * *p* < 0.05, *** *p* < 0.001.

**Table 3:** The moderating effect of interdependence and autonomy between emotional valence and perspective-taking.

		B	SE	t	LLCL	ULCL
Constant		2.76 ***	0.61	4.55	1.57	3.96
Emotional valence → Perspective-taking		−1.71	0.90	1.89	−3.49	0.07
Interdependence → Perspective-taking		−0.01	0.10	0.14	−0.20	0.18
Emotional valence × Interdependence → Perspective-taking		0.35 *	0.14	2.55	0.08	0.62
Autonomy → Perspective-taking		0.14	0.10	1.51	−0.04	0.33
Emotional valence × Autonomy → Perspective-taking		0.30 *	0.14	2.16	0.03	0.57
Conditional effect of emotion on perspective-taking at values of interdependence and autonomy						
Interdependence	Autonomy	B	SE	t	LLCI	ULCI
3.83	4.17	0.87 ***	0.23	3.75	0.42	1.33
3.83	5.33	1.22 ***	0.20	6.04	0.82	1.62
3.83	6.00	1.42 ***	0.24	5.98	0.95	1.89
5.00	4.17	1.28 ***	0.21	6.08	0.87	1.70
5.00	5.33	1.63 ***	0.15	10.92	1.34	1.93
5.00	6.00	1.83 ***	0.18	10.08	1.47	2.19
5.83	4.17	1.57 ***	0.26	6.01	1.06	2.09
5.83	5.33	1.92 ***	0.20	9.58	1.53	2.32
5.83	6.00	2.12 ***	0.22	9.74	1.69	2.55

Note. N = 307. Bootstrap sample size = 5000, LLCI = lower limit confidence interval, ULCI = upper limit confidence interval, * *p* < 0.05, *** *p* < 0.001.

**Table 4:** The association between emotional valence and creativity through perspective-taking: a moderated mediation analysis of interdependence and autonomy.

		B	SE	t	LLCI	ULCI
Constant		2.24 ***	0.17	12.90	1.90	2.58
Emotional valence → Creativity		0.30 *	0.14	2.17	0.03	0.57
Perspective-taking → Creativity		0.49 ***	0.04	11.12	0.41	0.58
Indices of partial moderated mediation						
		Index	BootSE		BootLLCI	BootULCI
Interdependence		0.17	0.07		0.03	0.32
Autonomy		0.15	0.07		0.01	0.31
Conditional indirect effect of emotional valence on creativity through perspective-taking based on the differences in interdependence and autonomy						
Interdependence	Autonomy	Effect	BootSE		BootLLCI	BootULCI
3.83	4.17	0.43	0.12		0.19	0.66
3.83	5.33	0.60	0.10		0.41	0.81
3.83	6.00	0.70	0.12		0.47	0.95
5.00	4.17	0.63	0.12		0.38	0.87
5.00	5.33	0.80	0.11		0.60	1.02
5.00	6.00	0.90	0.13		0.67	1.16
5.83	4.17	0.78	0.16		0.46	1.10
5.83	5.33	0.95	0.15		0.67	1.25
5.83	6.00	1.05	0.16		0.74	1.38

Note. N = 307. Bootstrap sample size = 5000, LLCI = lower limit confidence interval, ULCI = upper limit confidence interval, * *p* < 0.05, *** *p* < 0.001.

## Data Availability

The raw data supporting the conclusions of this article will be made available by the authors on request. Due to ethical restrictions imposed by the institutional review board, the raw data cannot be made publicly available. However, the data can be shared with researchers who meet the criteria for access to confidential data upon request.

## Abbreviations

The following abbreviations are used in this manuscript:

HIT	Human Intelligence Task
